# A novel method for peanut variety identification and classification by Improved VGG16

**DOI:** 10.1038/s41598-021-95240-y

**Published:** 2021-08-03

**Authors:** Haoyan Yang, Jiangong Ni, Jiyue Gao, Zhongzhi Han, Tao Luan

**Affiliations:** 1 College of Animation and Communication, Qingdao Agricultural University, Qingdao, 266109 Shandong China; 2 College of Science and Information Science, Qingdao Agricultural University, Qingdao, 266109 Shandong China

**Keywords:** Computer science, Mechanical engineering

## Abstract

Crop variety identification is an essential link in seed detection, phenotype collection and scientific breeding. This paper takes peanut as an example to explore a new method for crop variety identification. Peanut is a crucial oil crop and cash crop. The yield and quality of different peanut varieties are different, so it is necessary to identify and classify different peanut varieties. The traditional image processing method of peanut variety identification needs to extract many features, which has defects such as intense subjectivity and insufficient generalization ability. Based on the deep learning technology, this paper improved the deep convolutional neural network VGG16 and applied the improved VGG16 to the identification and classification task of 12 varieties of peanuts. Firstly, the peanut pod images of 12 varieties obtained by the scanner were preprocessed with gray-scale, binarization, and ROI extraction to form a peanut pod data set with a total of 3365 images of 12 varieties. A series of improvements have been made to VGG16. Remove the F6 and F7 fully connected layers of VGG16. Add Conv6 and Global Average Pooling Layer. The three convolutional layers of conv5 have changed into Depth Concatenation and add the Batch Normalization(BN) layers to the model. Besides, fine-tuning is carried out based on the improved VGG16. We adjusted the location of the BN layers. Adjust the number of filters for Conv6. Finally, the improved VGG16 model's training test results were compared with the other classic models, AlexNet, VGG16, GoogLeNet, ResNet18, ResNet50, SqueezeNet, DenseNet201 and MobileNetv2 verify its superiority. The average accuracy of the improved VGG16 model on the peanut pods test set was 96.7%, which was 8.9% higher than that of VGG16, and 1.6–12.3% higher than that of other classical models. Besides, supplementary experiments were carried out to prove the robustness and generality of the improved VGG16. The improved VGG16 was applied to the identification and classification of seven corn grain varieties with the same method and an average accuracy of 90.1% was achieved. The experimental results show that the improved VGG16 proposed in this paper can identify and classify peanut pods of different varieties, proving the feasibility of a convolutional neural network in variety identification and classification. The model proposed in this experiment has a positive significance for exploring other Crop variety identification and classification.

## Introduction

Peanut is one of the essential oil and economic crops globally, rich in nutrition and widely planted. The USDA forecasts World peanut production for 2020/21 at 47.79 million tons, of which China peanut production at 17.50 million tons^1^. The yield and quality of different peanut varieties are different. The identification of peanut varieties is an essential step in detecting seeds, phenotype collection and peanuts' scientific breeding. Peanut pod is the fruit of peanut and the morphological characteristics are an essential organ for testing DUS traits of peanut varieties^2^. The previous peanut variety identification work mainly includes two aspects: manual measurement and biochemical detection. However, the manual measurement of peanut pod variety identification has disadvantages such as slow identification speed, low accuracy and intense subjectivity, and the biochemical detection of peanut pod variety identification has disadvantages such as high cost and poor timeliness^3^. Therefore, there is an urgent need for a more accurate, economical and intelligent peanut variety identification method.

In recent years, research on crops based on image processing technology has made some progress and has been widely applied to many crops such as rice^4^, wheat^5^, soybean^6^. The application of image processing technology to peanut identification can effectively improve work efficiency and precision. Deng and Han^7^ used Fisher feature selection, SVM classification and K-means clustering analysis to extract five categories of 37 features from peanut pods and achieved 92.5% identification accuracy on the SVM model, and this work proved the feasibility of image processing techniques applied to peanut pod variety identification. Han et al.^8^ by extracting peanut kernels image morphology, texture, color appearance three classes, a total of 54 characteristics, using principal component analysis (PCA) for feature optimization and the neural network (ANN) and support vector machine (SVM) is used to identify the quality of peanut kernels, eventually be able to identify more than 95% of the imperfect, mildew, impurity, different varieties of different qualities, such as grain, the work will be successful image processing technology to broaden in the field of peanut quality. Yuan et al.^9^ use compose a specular like technology to obtain the health peanuts and mildew peanuts two kinds of image and USES support vector machine (SVM), partial least squares discriminant analysis (PLS-DA) soft pattern classification has nothing to do with clustering classifier (SIMCA) integrated classifier to classify, with 97.66% accuracy works to achieve the set highlights like technology application in peanut mildew prediction work. Traditional image processing methods have achieved some peanut identification achievements, but they need to extract many features in their work, which have defects such as intense subjectivity and insufficient generalization ability. Therefore, new technology is needed for intelligent feature extraction and classification of images.

Deep learning is a machine learning technology that has developed rapidly in recent years. It has become the essential tool for data processing in computer vision work and has been widely applied in many fields such as agricultural product classification^10^, synthetic speech recognition^11^, animal behavior analysis^12^, sensor signal recognition^13^ and COVID-19 detection^14,15^. The convolutional neural network (CNN) is a feedforward neural network that includes convolutional computation and has a deep structure, it is one of the main architectures of deep learning^16,17^. The convolutional neural network can automatically extract image features by simulating the biological vision mechanism. In the process of image identification, it can complete complex feature extraction^18,19^. Based on the deep learning method, the convolutional neural network is used to extract features and classify them in the peanut identification and classification task, which can often achieve good identification results. Zhang et al.^20^ used the neural network models AlexNet, GoogLeNet and the improved AlexNet to classify and recognize the five peanut pods' five levels, finally achieving an identification accuracy of 95.43%. Based on deep learning technology, Liu et al.^21^ used a convolutional neural network to recognize and classify hyperspectral images of healthy peanuts, damaged peanuts, and moldy peanuts and achieved an accuracy of 92.07%. In these works, the researchers have successfully used deep learning techniques to classify peanuts' quality and grade, But their study neglected to apply these new techniques to identify and classify peanut varieties. This paper will fill the gap.

This paper, based on deep learning technology, Brings some novel improvements to VGG16. Remove the F6 and F7 fully connected layers of VGG16. Add Conv6 and Global Average Pooling Layer. The three convolutional layers of conv5 are changed into Depth Concatenation. Then, add The BN layers to the model. Besides, fine-tuning is carried out based on the improved VGG16. We adjusted the location of the BN layers. Adjust the number of filters for Conv6. The advantages and characteristics of the improved VGG16 model are analyzed, and the influence of different network improvement methods on the training effect is compared. Finally, the improved VGG16 model's test effect on the peanut pod data set was compared with other classical models to verify its superiority. The improved VGG16 model was applied to the identification and classification of seven maize varieties, in the same way, the supplementary experiments were carried out to prove the robustness and versatility of the improved VGG16 model.

The remainder of the paper is structured as follows: “Materials and methods” section introduces the experimental materials and methods. “Results and analysis” section makes a detailed analysis of the experimental results. “Discussion” section discusses some problems encountered in the research process. Finally, “Conclusions” section summarizes the research.

## Materials and methods

### Materials

#### Peanut sample preparation

A total of 12 peanut varieties were used in the experiment, all of which were retained by farmers. The experimental peanut collection areas mainly include Hebei, Qingdao, Rizhao and Laiyang of Shandong. Peanut varieties are mainly large peanut varieties in north China. The peanut samples were all healthy and undamaged peanuts. The names and origins of the 12 varieties of peanuts are shown in Table 1.

**Table 1:** Experimental materials for peanut variety identification.

Code	Variety	Source	Code	Variety	Source
1	101 Huasheng	Laiyang	7	Qinghua 6	Laiyang
2	Huayu 22	Laiyang	8	Tianfu 3	Hebei
3	Huayu 25	Laiyang	9	Yihua 2	Hebei
4	Lainong 13	Laiyang	10	Yihua 4	Hebei
5	Luhua 9	Rizhao	11	Yihua 5	Hebei
6	Luhua 11	Qingdao	12	Zhongnong 108	Hebei

#### Image acquisition system

**Figure 1:**
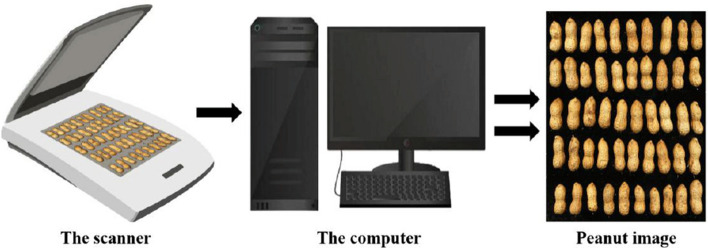
The workflow of peanut image collection.

A scanner was used to collect peanut pod images. When using the scanner, the scanner’s cover plate is fully open to scanning background black. During image collection, the peanut is uniformly placed on the scanner in a fixed order for image scanning. The peanut pod image obtained by the scanner is transferred to the computer for further image processing. The schematic diagram of the peanut pod image collection is shown in Fig. 1. The scanner used in the experiment is Canon Canoscan 8800F, flat CCD scanner, optical resolution of 4800 dPix9 600 dpi; The maximum resolution is 19,200 dpi and the scanning range is 216 MMX 297 mm. The computer used to store the images was a Lenovo IdeaCentre Kx 8160.

### Methods

#### Data preprocessing

**Figure 2:**
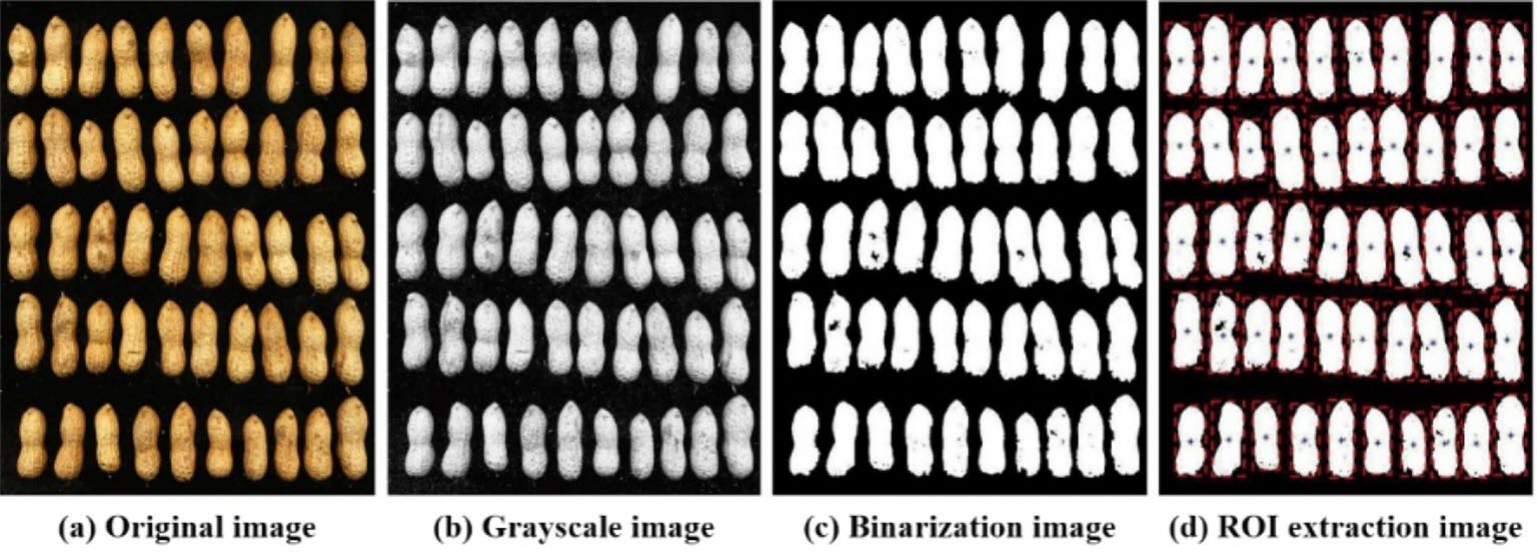
The workflow of peanut image segmentation.

To generate individual images of 12 varieties of peanut pods, it is necessary to preprocess the images of peanut pods obtained by the scanner. The image segmentation process is shown in Fig. 2. Figure 2a is the original image of the peanut output from the scanner. Figure 2b is obtained after gray processing of the original image. The binarization image is obtained by image binary processing, expansion and threshold segmentation, as shown in Fig. 2c. ROI extraction was carried out. By retrieving the contour of the connected region, the area of the connected region was obtained. The contour box of a single peanut pod was selected to get Fig. 2d. Finally, the single peanut pod selected in the box is mapped to the original image and the single peanut pod image is extracted and stored.

**Figure 3:**
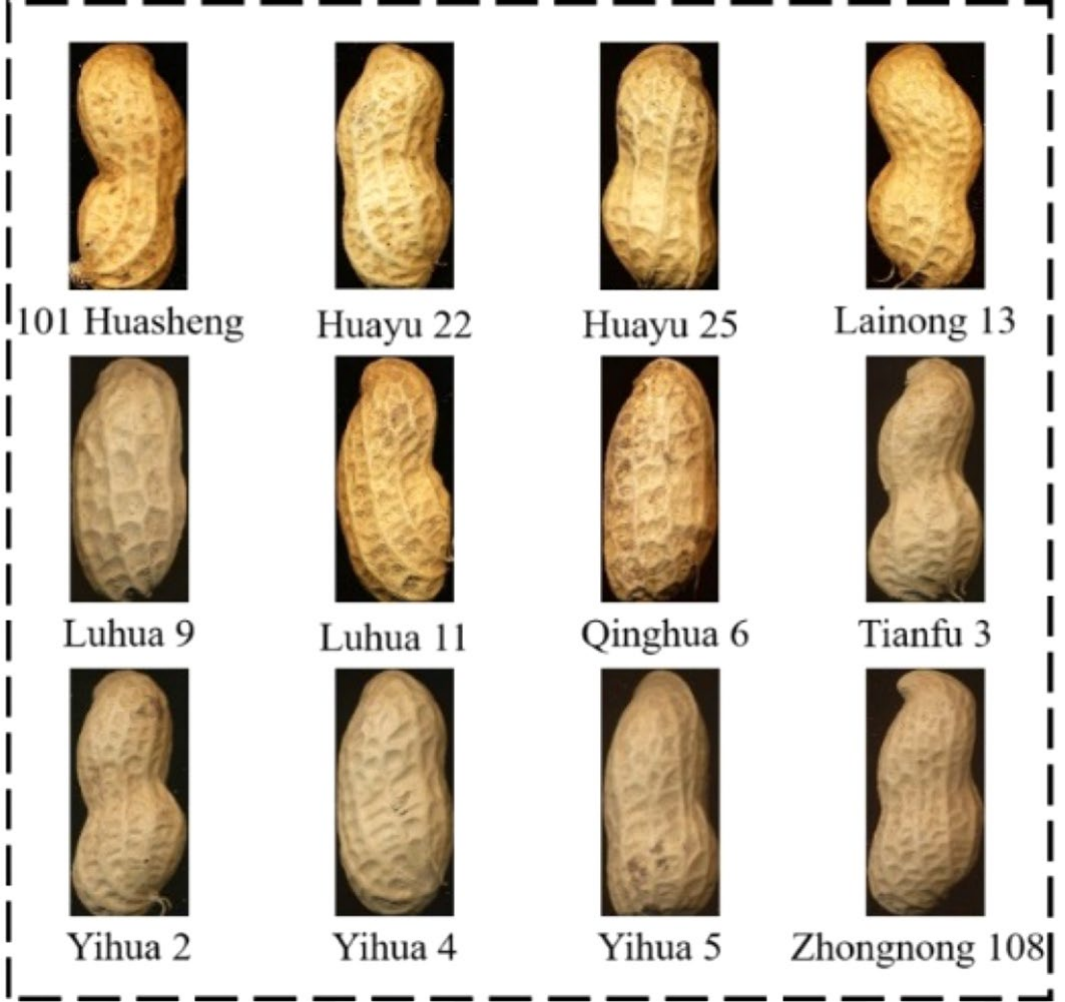
Images of 12 peanut varieties.

By the above image segmentation method, all the images of peanut pods of 12 varieties obtained by the scanner were segmented into single images and stored in 12 categories according to the species. The single images of peanut pods of 12 varieties obtained are shown in Fig. 3.

Through image segmentation processing, 12 varieties of 3365 images of peanut pods data set were received. To meet the requirements of deep learning training, the peanut pods data set according to the proportion of 8:1:1 were randomly divided into the training set, validation set and test set. Twelve varieties of peanut pods are divided into 12 categories, and each type of uniform distribution gets peanut pod identification data set. Finally completed the identification of peanut pod data sets is shown in Table 2.

**Table 2:** Peanut pod identification data set.

Code	Label	Train set	Validation set	Test set	Total
1	101 Huasheng	234	29	29	292
2	Huayu 22	234	29	29	292
3	Huayu 25	228	29	29	286
4	Lainong 13	228	29	29	286
5	Luhua 9	224	28	28	280
6	Luhua 11	115	14	14	143
7	Qinghua 6	238	30	30	298
8	Tianfu 3	240	30	30	300
9	Yihua 2	239	30	30	299
10	Yihua 4	238	30	30	298
11	Yihua 5	233	29	29	291
12	Zhongnong 108	240	30	30	300
0	All	2691	337	337	3365

#### Network improvement

Convolutional Neural Network (CNN) improves BP Neural Network, which is often used in computer vision tasks due to its ability to represent local operations' abstract hierarchical representation^22^. The network model comprises the convolutional layer, the Pooling layer and the fully connected layer. The convolutional layer comprises several convolution units, and the backpropagation algorithm optimizes each convolution unit's parameters. The function of the convolutional layer is to extract various features of the input image. The Pooling layer is an integral part of the convolutional neural network. It is to de-sample the data. Its function is to reduce the amount of data to be processed at the next layer, reduce the number of parameters and prevent network overfitting. Each neuron in the fully connected layer is fully connected with all neurons in the previous layer and the fully connected layer can integrate local information with category discrimination in the convolutional layer or pooling layer.

VGG16 is a convolutional neural network model developed by the Visual Geometry Group (VGG) of the University of Oxford and the winner of the 2014 ILSVRC object identification algorithm^23^. The critical work of VGG16 is to demonstrate that extending the depth of the network can improve the performance of the network in certain situations. Compared with the classic AlexNet, VGG16's improvement lies in the use of multiple 3 × 3 convolution cores to replace the larger convolution cores (11 × 11, 7 × 7, 5 × 5), which can broaden the depth of the network to improve the network performance effectively, and the use of smaller convolution cores can also reduce the number of network parameters. The VGG16 network model comprises 13 convolutional layers, three fully connected layers and five pooling layers.

**Figure 4:**
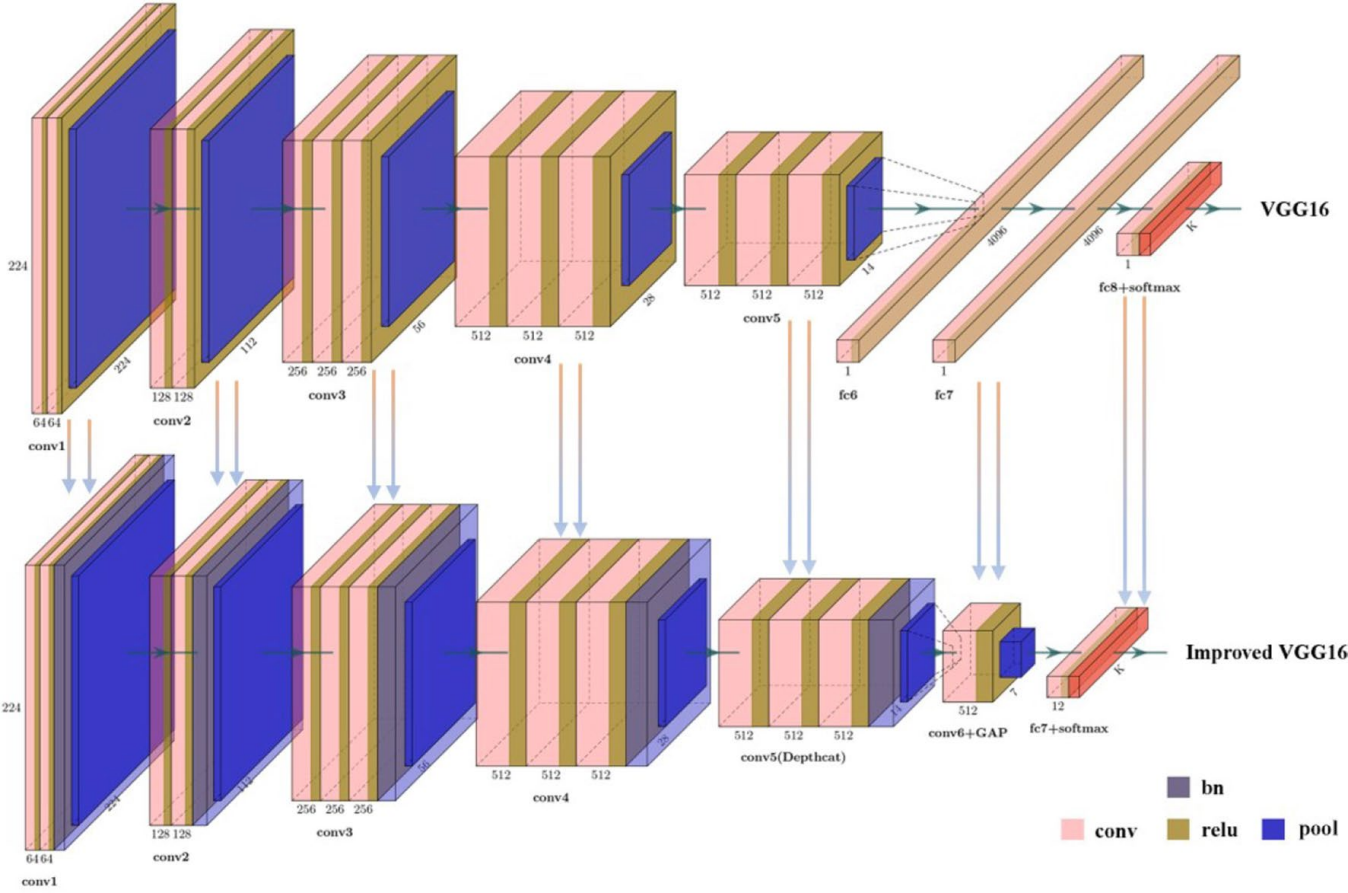
Network structure diagram of VGG16 and Improved VGG16.

VGG16 continues the characteristics of the classical network's simple structure, expands the network's depth through the flexible use of 3 × 3 convolution and successfully improves network performance. However, the VGG16 model also has some drawbacks in the application. First, the fully connected layer has many parameters, which occupy much memory and consume many computing resources, making the VGG16 model encounter obstacles in the front-end deployment. Secondly, the network model structure is single, and its performance is weak compared with some sophisticated advanced networks. Moreover, VGG16 lacks an effective method to prevent gradients' disappearance and problems such as slow convergence speed and gradient explosion are likely to occur in the model's training. Aiming at the defects of the VGG16, this paper improved the VGG16 model by drawing on advanced network models such as ResNet, SqueezeNet and DenseNet. The improved VGG16 model consists of 14 convolutional layers, five BN layers, six pooling layers and one fully connected layer. The network structures of VGG16 and Improved VGG16 are shown in Fig. 4.

The main improvement methods of the improved VGG16 model are as follows.

Remove the F6 and F7 fully connected layers of VGG16. Add Conv6 and Global Average Pooling Layer(GAP). The two fully connected layers FC6 and FC7, in the VGG16, willfully connect each neuron with all the neurons in the previous layer, thus generating a considerable number of parameters and occupying many computing resources. Therefore, these two fully connected layers need to be discarded. GAP is a new idea proposed by M. Lin et al. (2014), which can replace the fully connected layer, and it has been proved by experiments that GAP can reduce the number of parameters, the amount of calculation and the amount of overfitting in the model^24^. GAP can calculate the mean value of the pixel points in each feature map, output a feature point, and fuse these feature points into feature vectors and input them to the Softmax layer, thus reducing the number of parameters, the amount of calculation and the over-fitting. Besides, GAP can output a feature graph for each category, which directly endows features with real meaning and connects each category and feature graph more intuitively. As more and more researchers have confirmed GAP's function, many advanced network models, such as GoogLeNet, ResNet, SqueezeNet and DenseNet, have introduced a GAP. SqueezeNet^25^ added a convolutional layer with a convolution kernel size of 1 × 1 before the GAP to balance input and output channel size. This operation again reduced the number of parameters and computation in the model and significantly accelerated the speed. Therefore, in this paper, a convolutional layer Conv6 with a convolution kernel size of 1 × 1 was placed in front of the added GAP to optimize the model further. The number of filters on the model performance was analyzed by setting various filters (128/256/512) during model construction.

**Figure 5:**
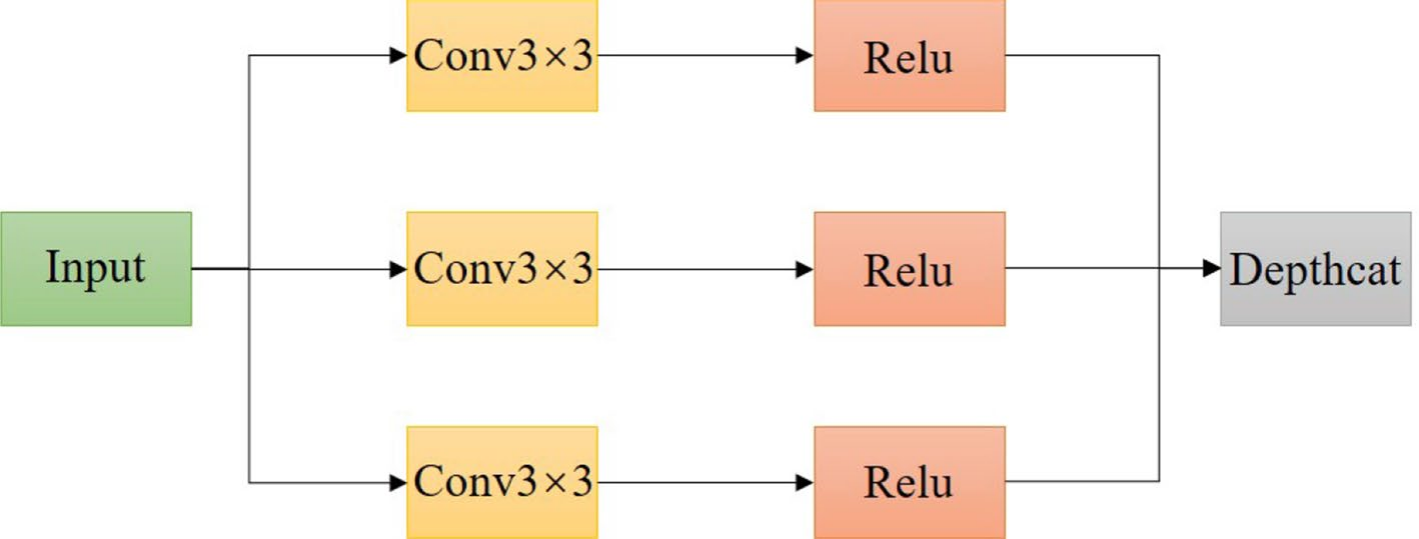
Conv5 structure diagram.

Conv5 of the VGG16 network model was changed to a deeply tandem group. VGG16 model continues the simple network structure of classical models such as Lenet5 and AlexNet. Although the network depth has been further expanded to improve the performance, compared with some sophisticated advanced networks, the network model structure is single and the model complexity is too low, making it challenging to deal with some complex tasks. In this paper, using the Inception structure of GoogLeNet^26^ for reference, the three convolutional layers of the VGG16 network model Conv5 are transformed into a deeply tandem group. In this structure, the upper of the input characteristics will all pass to each convolutional layer. It will not produce the wastage of the decreasing step by step, and This can increase the complexity of the model to a certain extent and improve the width of the network, make conv5 learn more characteristics, improve the identification accuracy of the network and have the effect of model optimization. The improved Conv5 is shown in Fig. 5.

Add the BN layers. Batch Normalization process refers to pulling the input values in neural network neurons back to the standard normal distribution, where the mean is 0 and the variance is 1. This operation will place the input values in the input-sensitive areas of non-linear function pairs. In this way, small changes in the input value can substantially impact the loss function, and the gradient can be increased to prevent the problem of gradient disappearance. Besides, the convergence speed and training speed of the model can be significantly accelerated. The Batch Normalization algorithm^27^ is as follows:

Batch input *m* samples: $x_{1} \sim x_{m}$, then the mean value of batch data $\mu$ as:

1 $$ \mu = \frac{1}{m}\sum\limits_{i = 1}^{m} {x_{i} } $$

Calculate the variance of batch data $\sigma^{2}$ as:

2 $$ \sigma^{2} = \frac{1}{m}\sum\limits_{i = 1}^{m} {\left( {x_{i} - \mu } \right)}^{2} $$

Normalize the data:

3 $$ \hat{x}_{i} = \frac{{x_{i} - \mu }}{{\sqrt {\sigma^{2} + \in } }} $$

Dimension transformation and offset:

4 $$ y_{i} = \gamma \hat{x}_{i} + \beta = BN_{\gamma ,\beta } \left( {x_{i} } \right) $$

It has been proven that adding The BN layers to network models has a significant benefit, but there is no definitive answer to where it will be in the network. When the BN was first described in 2015, it was added to the authors' front of the ReLU layer. Nevertheless, as the process of BN has been used in more and more studies, some researchers have suggested that it does better to have The BN layer after the ReLU layer. Kohlhepp B et al. stated in their studies that placing the BN layer in front of the ReLU layer will sometimes hurt the model while putting the BN layer after the ReLU layer will have positive effects such as improving accuracy and reducing loss^28^. Therefore, there are two different methods of adding The BN layer to the VGG16 model in this paper. The first method is to place the BN layer between the Conv layer and the ReLU layer of the model, resulting in Model 1. The second method is to place the BN layer between the ReLU layer and the Pooling layer of the model and obtain Model 2. After training tests, compare the different placement of the BN layers on the model's performance. The specific network parameters of Model 1 and Model 2 are shown in Table 3.

**Table 3:** Three kinds of network model comparison.

VGG16	Model1	Model2
Input (224*224*3)	Input (224*224*3)	Input (224*224*3)
Conv1_1_64	Conv1_1_64	Conv1_1_64
Conv1_2_64	Batch Normalization1	Conv1_2_64
MaxPooling1	Conv1_2_64	Batch Normalization1
	MaxPooling1	MaxPooling1
Conv2_1_128	Conv2_1_128	Conv2_1_128
Conv2_2_128	Batch Normalization2	Conv2_2_128
MaxPooling2	Conv2_2_128	Batch Normalization2
	MaxPooling2	MaxPooling2
Conv3_1_256	Conv3_1_256	Conv3_1_256
Conv3_2_256	Batch Normalization3	Conv3_2_256
Conv3_3_256	Conv3_2_256	Conv3_3_256
MaxPooling3	Conv3_3_256	Batch Normalization3
	MaxPooling3	MaxPooling3
Conv4_1_512	Conv4_1_512	Conv4_1_512
Conv4_2_512	Batch Normalization4	Conv4_2_512
Conv4_3_512	Conv4_2_512	Conv4_3_512
MaxPooling4	Conv4_3_512	Batch Normalization4
	MaxPooling4	MaxPooling4
Conv5_1_512	Conv5_1_512	Conv5_1_512
Conv5_2_512	Batch Normalization5_1	Conv5_2_512
Conv5_3_512	Conv5_2_512	Conv5_3_512
MaxPooling5	Batch Normalization5_2	Depth Concatenation
	Conv5_3_512	Batch Normalization5
FC6	Batch Normalization5_3	MaxPooling5
	Depth Concatenation	
FC7	MaxPooling5	Conv6_512
	Conv6_512	GAPooling6
FC-1000	GaPooling6	
	FC-12	FC-12
SoftMax	SoftMax	SoftMax

## Results and analysis

### Model training results

The experiment was carried out with Matlab2020a software under the Windows10 system. The peanut pod data sets were imported into VGG16, Model1 and Model2, respectively. The parameters were set as: Image Input Size: 224 × 224 × 3;Mini BatchSize: 32; Initial Learn Rate: 1e-4; Validation Frequency: 64. Then start training. All models finished training after 50 Epochs, and the absolute accuracy of the models was the average accuracy of the three times of model training. Table 4 shows the final accuracy tables of VGG16, Model1 and Model2. It can be seen from Table 4 that VGG16 achieves an average accuracy of 88.39% on the validation set, while Model1 achieves an average accuracy of 94.94%, 6.55% higher than VGG16. Model2 achieves the highest average accuracy of 97.62%, 2.68% higher than Model1. We see similar results in the test set. VGG16 achieves an average accuracy of 87.80%, while Model1 achieves an average accuracy of 93.90%, which is 6.10% higher than VGG16. Model2 achieves the highest average accuracy of 96.7%, which is 2.80% higher than Model1. The results show that Our improved network model is effective, and the identification and classification ability of the improved VGG16 is better than VGG16. The average Accuracy of Model 2 is higher than that of Model 1, which indicates that placing the BN layer between the ReLU layer and the Pooling layer of the model is reasonable and successful. Based on the above experimental results and analysis, we determine that Model2 is the final model constructed for this experiment, which we will call "Our Model" in the following content.

**Table 4:** Accuracy of the three kinds of network.

Models	Validation accuracy(%)				Test accuracy(%)			
	First	Second	Third	Average	First	Second	Third	Average
VGG16	89.58	88.39	87.20	88.39	87.20	87.80	88.40	87.80
Model1	94.05	95.83	94.94	94.94	93.50	93.50	94.70	93.90
Model2	97.32	97.62	97.92	97.62	96.40	96.70	97.00	96.70

In the construction of Our Model, the number of filters of Conv6 was set to 512. To investigate the influence of the number of filters of Conv6 on the model performance, the number of filters of Conv6 was set to 128/256 respectively for fine-tuning the model. The peanut pods data set was imported into the fine-tuning model twice, with the parameters set as Image Input Size: 224 × 224 × 3; Mini BatchSize: 32; Initial Learn Rate: 1e-4; Validation Frequency: 64. Then start training. All models finished training after 50 Epochs and the absolute accuracy of the models was the average accuracy of the three times of model training. Table 5 shows the final accuracy table of the fine-tuning model. Model(128) and Model(256) are fine-tuning models with 128 and 256 filters in conv6, respectively and model (512) is Our Model with 512 filters in conv6. It can be seen from Table 5 that the average Accuracy of Our Model on the validation set and the test set is 97.62% and 96.70%, respectively, both of which are the highest average accuracy among the three models. The experimental results show that Our Model with 512 filters in conv6 is the optimal model for this experiment. Also, the accuracy of Model(256) is higher than that of the model (128) on both the validation set and the test set, which indicates that in this experiment, with the orderly increase of the number of filters in Conv6, the identification accuracy of the model is improved regularly.

**Table 5:** Accuracy of the fine-tuning network.

Models	Validation accuracy(%)				Test accuracy(%)			
	First	Second	Third	Average	First	Second	Third	Average
Model (128)	96.73	96.13	97.32	95.73	96.10	96.10	95.80	96.00
Model (256)	97.62	97.32	97.62	97.52	96.10	96.40	96.10	96.20
Model (512)	97.32	97.62	97.92	97.62	96.40	96.70	97.00	96.70

### Comparison of model performance

**Figure 6:**
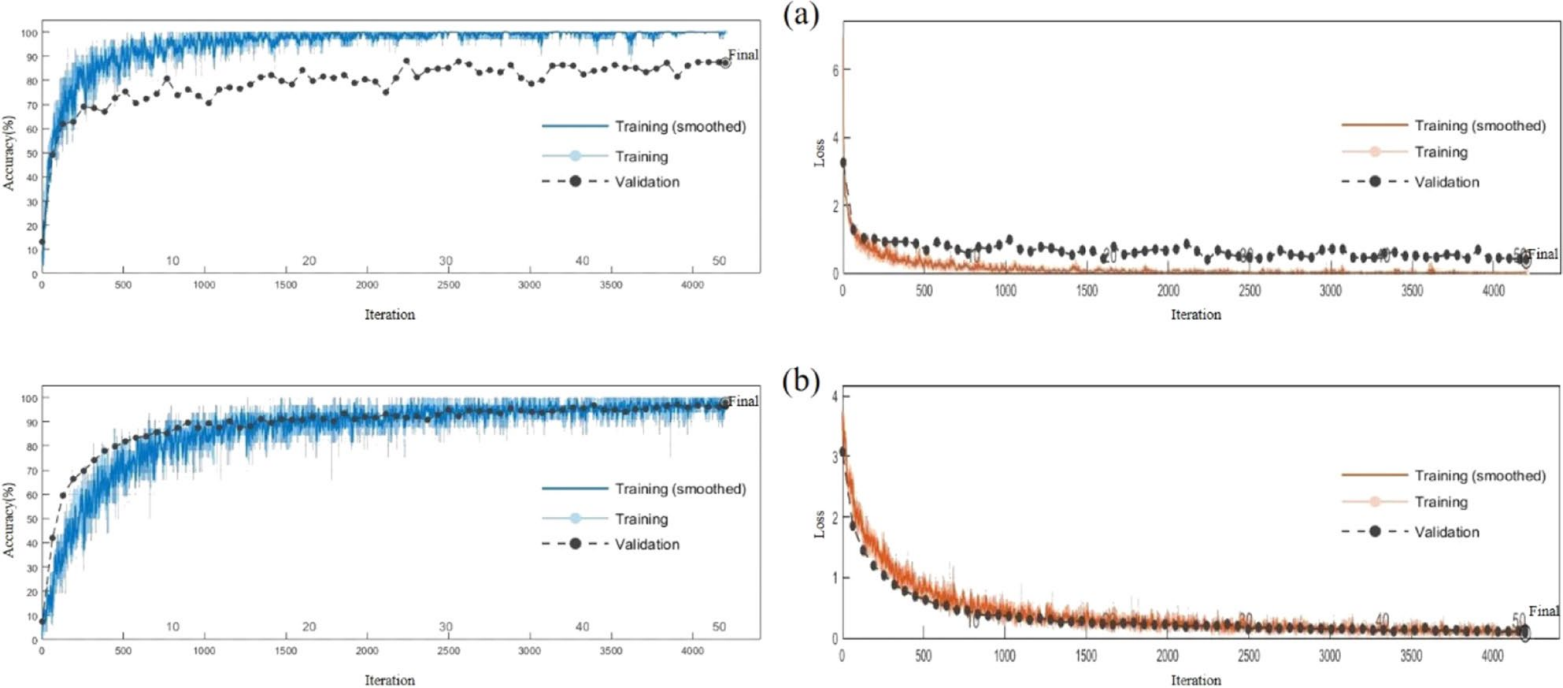
Model training result comparison.

Figure 6 shows the training result comparison between VGG16 and Our Model, Fig. 6a shows VGG16 and Fig. 6b shows Our Model. The accuracy training chart's blue curve is the training accuracy curve and the black dotted line is the validation accuracy curve. In the loss error training diagram, the red curve is the training loss rate curve, while the black dotted line is the validation loss rate curve. Figure 6 shows that VGG16 model after 50 Epochs of training, the training accuracy increased from 90 to 100%. However, it cannot effectively improve the validation set. Model validation accuracy has been hovering between 70 and 90% and verifying the accuracy of 87.20%. Its validation loss rate and loss rate of training also have a large gap, suggesting that VGG16 produced over the fitting phenomenon. VGG16 model can not finish the peanut pod identification task successfully. Our Model's training process shows that after 50 Epochs training of Our Model, both the training accuracy and validation accuracy reached more than 90%, the validation accuracy finally reached 97.92% and the loss rate gradually decreased to 0. These phenomena indicate that Our Model has an excellent performance in the identification and classification of peanut pods. Also, the validation accuracy of Our Model increases gradually with the improvement of training accuracy, and there is no over-fitting or under-fitting phenomenon, indicating that Our Model has an excellent ability to resist over-fitting and under-fitting. Besides, the training accuracy rate of Our Model rapidly increases to 90% after 15 Epochs, which indicates that Our Model has a high convergence speed.

**Figure 7:**
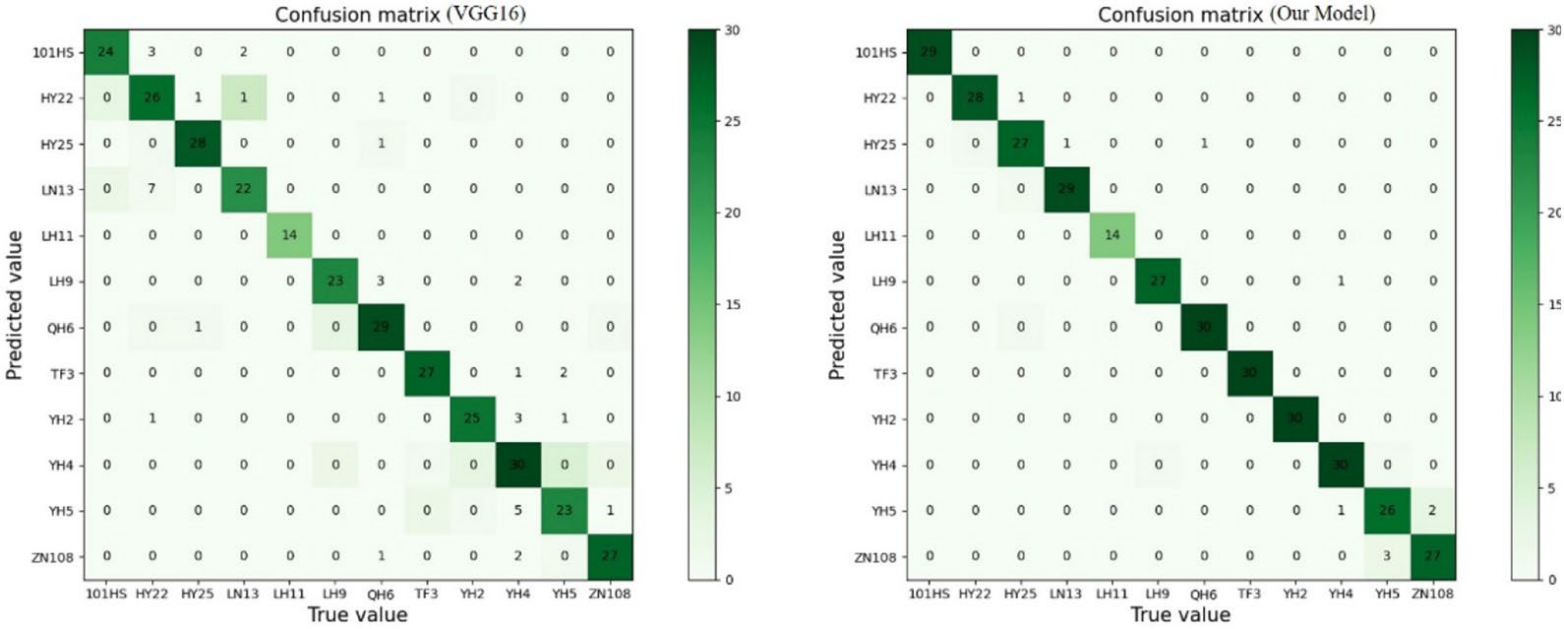
Model confusion matrix comparison.

Figure 7 shows the confusion matrix comparison between VGG16 and Our Model. The confusion matrix is an important index to measure the performance of the model. The sum of each row of the confusion matrix represents the actual sample number of the predicted category. For example, the sum of the first row of VGG16 and Our Model confusion matrix in Fig. 7 is 29, which means that the actual sample number of 101 Huasheng tested in the two models is 29. Confuse the sum of each column of the matrix representation is predicted for a sample size of the class. For example, in Fig. 7, VGG16 confuse the sum of the second column of the matrix to 37; this means that in the process of the VGG16 model test, there are 37 samples were predicted to became Huayu 22; Our Model confuse the sum of the second column of the matrix is 28, this means that in Our Model of the test process, there are 28 samples were predicted to become the Huayu 22, by comparing the Huayu 22 actual sample size (29), It was found that VGG16 generated 11 misidentification and three omissions in the identification of Huayu 22 samples. In contrast, Our Model generated only one omission in identifying Huayu 22 samples and no misidentification was generated. Finally, by comparing the average test accuracy between VGG16 and Our Model of 87.80% and 96.70%, combined with the analysis of the number of correct identification, wrong identification and missing identification of each category of the two models, we determined that Our Model played a better role than the VGG16 in the peanut pod identification work.

Kappa is a consistency check coefficient based on the model confusion matrix, displaying identification accuracy and measuring model performance. Kappa's calculation is between -1 and 1, but usually, Kappa falls between 0 and 1. The corresponding relationship between Kappa and consistency is 0.21–0.40 "acceptable" consistency, 0.41–0.60 "medium" consistency, 0.61–0.80 "large" consistency and above 0.81 "almost perfect" consistency^29^.

Kappa's calculation formula is as follows:

5 $$ Kappa = \frac{Observed \, Accuracy - Expected \, Accuracy}{{1 - Expected \, Accuracy}} $$

According to the above formula, the Kappa of the VGG16 test result is 0.87 and the Kappa of Our Model test result is 0.97. It can be seen that Our Model achieves better consistency than VGG16, and the predicted results are almost entirely consistent with the actual identification results.

**Figure 8:**
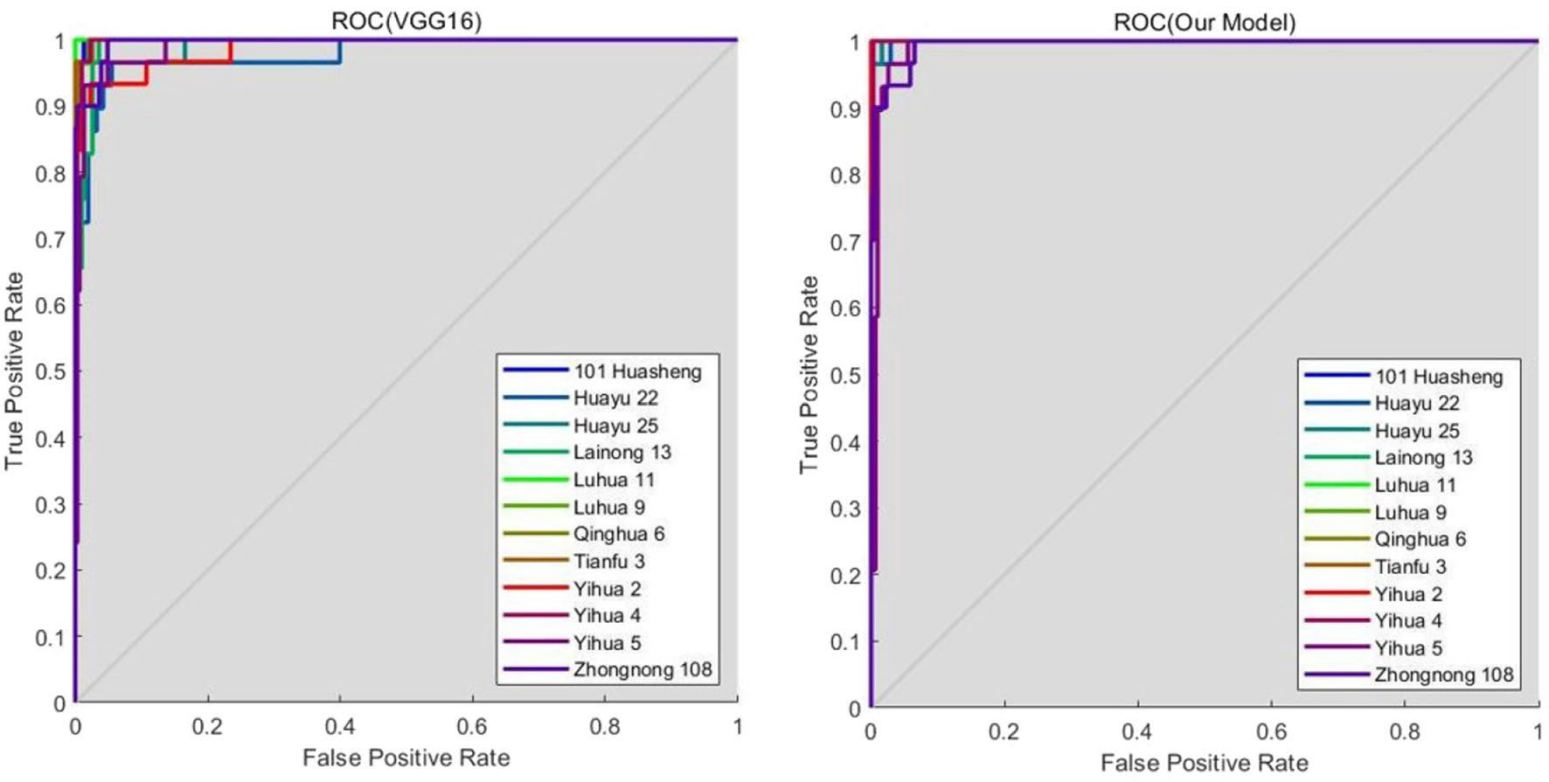
Model ROC curve comparison.

Figure 8 is the ROC curve comparison of VGG16 and Our Model test results. ROC curve^30^ is also called the susceptibility curve, is to reflect the sensitivity and specificity of the continuous variable comprehensive index, ROC curve by using a continuous variable, set out several different thresholds, it is concluded that sensitivity and specificity, sensitivity to ordinate and abscissa (1—specificity) to draw into a curve, the greater the area under the curve (AUC), the higher the accuracy. The point closest to the upper left of the coordinate graph is the critical point with high sensitivity and specificity. According to the ROC curves of the two models, it can be seen that the ROC curve of Our Model is closer to the upper left corner and the area under the curve is more extensive, indicating that Our Model has excellent performance.

### Comparison with classical models

To further explain Our Model's superiority, this section added an analysis of the training results of classic network models AlexNet, VGG16, GoogLeNet, ResNet18, ResNet50, SqueezeNet, DenseNet201 and MobileNetv2. The peanut pod data sets were imported into the above models for training and the model sizes and training parameters were shown in Table 6.

**Table 6:** Model size and training parameters.

Model	Layer	Size/MB	Mini batch size	Initial learn rate	Validation frequency	Image input size
AlexNet	25	227	32	1e−4	64	227 × 227 × 3
VGG16	41	515	32	1e−4	64	224 × 224 × 3
GoogLeNet	144	27	32	1e−4	64	224 × 224 × 3
ResNet18	71	44	32	1e−4	64	224 × 224 × 3
ResNet50	177	96	32	1e−4	64	224 × 224 × 3
SqueezeNet	68	4.6	32	1e−4	64	227 × 227 × 3
DenseNet201	708	77	32	1e−4	64	224 × 224 × 3
MobileNetv2	154	13	32	1e−4	64	224 × 224 × 3
Our Model	45	52.3	32	1e−4	64	224 × 224 × 3

**Figure 9:**
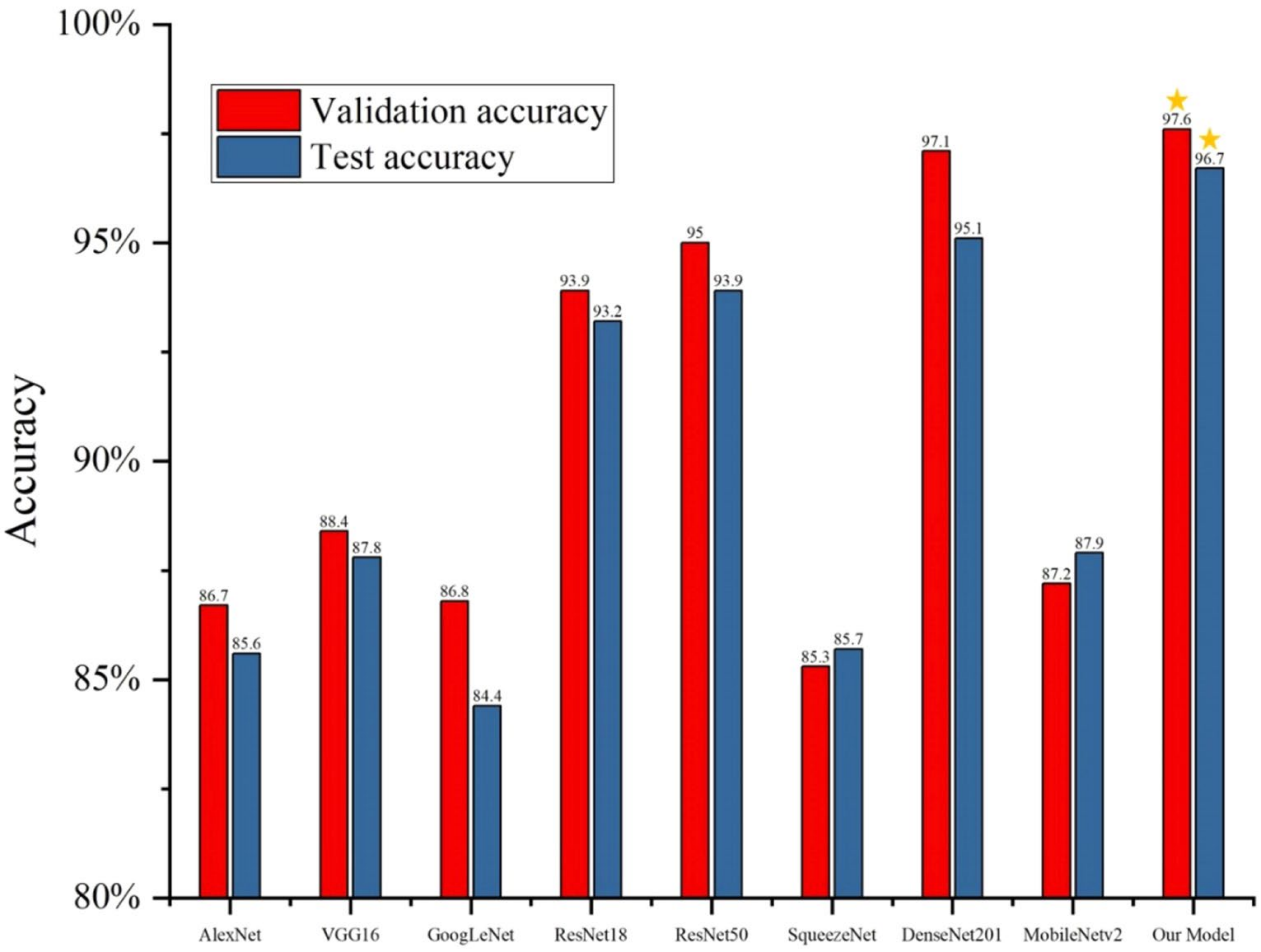
Average accuracy of all models.

All models finished training after 50 Epochs. Figure 9 shows the average validation accuracy and average test accuracy of all models. As shown in Fig. 9, the average Accuracy of AlexNet, VGG16, GoogLeNet, SqueezeNet and MobileNetv2 models on the validation set were all between 80 and 90% average accuracy of SqueezeNet was the lowest, which was 85.3%. The advanced ResNet18, Resnet50 and DenseNet201 exceeded the average accuracy of 90%. DenseNet201 achieving a good score of 97.1%, but still behind Our Model's average accuracy of 97.6%, which was 0.5% lower than Our Model's average accuracy. In the test set, the average accuracy of AlexNet, VGG16, GoogLeNet, SqueezeNet and MobileNetv2 models remained between 80 and 90%, with GoogLeNet having the lowest average accuracy of 84.4%. ResNet18, Resnet50 and DenseNet201 again performed well, with an average accuracy of over 90%. DenseNet201 achieving an average accuracy of 95.1%, which was still 1.6% lower than Our Model's average accuracy of 96.7%. The experimental results show that all the models play an excellent role in identifying and classifying peanut pods. Usually, Which can easily lead to overfitting if the training model is too deep. Therefore, Resnet18, Resnet50 and Densenet201 are inferior to Our Model in this fine identification task. Our Model has the highest accuracy and the most robust identification ability in both the validation set and the test set.

Accuracy, Precision, Recall and F1-score were introduced in this paper to evaluate each model's performance comprehensively. Accuracy is one of the most common evaluation criteria. Accuracy represents the proportion of all correctly identified samples to the total. Accuracy is a very intuitive evaluation index, but sometimes it can be deceptive. When the number of samples is unbalanced, the value of accuracy tends to favor more samples. Therefore, based on accuracy's evaluation, more indicators need to be evaluated to measure the model's performance. Precision is one of the indicators that can represent the correct prediction ability of the model. Its significance is the proportion of correctly predicted samples in the model's total predicted results. The recall is also known as detection rate, which refers to the proportion of correctly predicted samples in the total of actual samples. F1-score is a comprehensive evaluation index, which is the synthesis of Precision and Recall indexes and its value range is 0–1.1 represents the optimal output of the model and 0 represents the worst output of the model. Accuracy, Precision, Recall, F1-score are calculated as follows:

Start by defining four basic metrics. The actual value is positive and the sample predicted by the model to be positive is $TP$. The actual value is positive and the sample predicted as unfavorable by the model is $FN$. The actual value is negative and the model's sample to be positive is denoted as $FP$. The actual value is negative and the number of negative predicted by the model is $TN$. Then the calculation formulas of Accuracy, Precision(P), Recall(R) and F1-Score are:

6 $$ Accuracy = \frac{TP + TN}{{TP + TN + FP + FN}} $$

7 $$ P = \frac{TP}{{TP + FP}} $$

8 $$ R = \frac{TP}{{TP + FN}} $$

9 $$ F1 - Score = \frac{2PR}{{P + R}} $$

Table 7 shows the model evaluation indexes of AlexNet, VGG16, GoogLeNet, ResNet18, Resnet50, SqueezeNet, DenseNet201, MobileNetv2 and Our Model in the peanut pods identification and classification task. Table 7 shows that the average accuracy of Our Model is 99.5%, 0.1–1.8% higher than other models. The average Precision of Our Model is 97.2%, 0.7–10.5% higher than other models. The average Recall of Our Model is 97.2%, 0.9–10.5% higher than other models. The average F1-Score of Our Model is 97.2%, 0.8–11.1% higher than other models. Model evaluation results showed that Our Model was superior to other models in the comprehensive evaluation of performance indexes such as Accuracy, Precision, Recall and F1-Score in the identification process of 12 varieties of peanut pods.

**Table 7:** Model performance evaluation(Class names are indicated by their initials).

Measures	101HS	HY22	HY25	LN13	LH11	LH9	QH6	TF3	YH2	YH4	YH5	ZN108	Average
**MODEL: AlexNet**													
Accuracy(%)	98.5	96.1	98.8	96.4	99.4	98.8	99.4	99.4	96.4	97.0	95.3	97.0	97.7
Precision(%)	100	80.8	90.3	75.8	92.9	92.9	93.8	96.7	82.1	79.4	68.6	95.5	87.4
Recall(%)	82.8	72.4	96.6	86.2	92.9	92.9	100	96.7	76.7	90.0	82.8	70.0	86.7
F1-Score(%)	90.6	76.4	93.3	80.7	92.9	92.9	96.8	96.7	79.3	84.4	75.0	80.8	86.7
**MODEL: VGG16**													
Accuracy(%)	98.5	95.8	99.1	97.0	100	98.5	97.9	99.1	98.5	96.1	97.3	98.8	98.1
Precision(%)	100	70.3	93.3	88.0	100	100	82.9	100	100	69.8	88.5	96.4	90.8
Recall(%)	82.8	89.7	96.6	75.9	100	82.1	96.7	90.0	83.3	100	79.3	90.0	88.9
F1-Score(%)	90.6	78.8	94.9	81.5	100	90.2	89.3	94.7	90.9	82.2	83.6	93.1	89.2
**MODEL: GoogLeNet**													
Accuracy(%)	99.4	97.6	98.5	97.3	99.7	98.5	98.5	97.6	95.0	97.9	95.5	97.0	97.7
Precision(%)	100	83.9	96.2	88.5	93.3	96.0	85.7	92.3	66.7	89.7	69.4	95.5	88.1
Recall(%)	93.1	89.7	86.2	79.3	100	85.7	100	80.0	86.7	86.7	86.2	70.0	87.0
F1-Score(%)	96.4	86.7	90.9	83.6	96.5	90.6	92.3	85.7	75.4	88.2	76.9	80.8	87.0
**MODEL: ResNet18**													
Accuracy(%)	99.7	98.5	99.7	98.8	100	100	99.7	99.1	99.1	99.4	96.7	96.7	99.0
Precision(%)	100	87.5	100	96.3	100	100	96.8	93.5	93.5	96.7	78.1	88.0	94.2
Recall(%)	96.6	96.6	96.6	89.7	100	100	100	96.7	96.7	96.7	86.2	73.3	94.1
F1-Score(%)	98.3	91.8	98.3	92.9	100	100	98.4	95.1	95.1	96.7	82.0	80.0	94.1
**MODEL: ResNet50**													
Accuracy(%)	99.1	97.6	99.1	98.8	99.7	99.7	99.7	100	100	99.4	97.9	98.2	99.1
Precision(%)	96.4	81.8	96.4	100	93.3	100	96.8	100	100	96.7	86.7	90.0	94.8
Recall(%)	93.1	93.1	93.1	86.2	100	96.4	100	100	100	96.7	89.7	90.0	94.9
F1-Score(%)	94.7	87.1	94.7	92.6	96.5	98.2	98.4	100	100	96.7	88.2	90.0	94.8
**MODEL: SqueezeNet**													
Accuracy(%)	99.4	96.1	98.8	96.7	99.1	98.8	98.8	98.8	96.1	97.0	95.5	96.7	97.7
Precision(%)	100	78.6	90.3	87.5	82.4	87.5	88.2	96.4	77.4	81.3	70.6	100	86.7
Recall(%)	93.1	75.9	96.6	72.4	100	100	100	90.0	80.0	86.7	82.8	63.3	86.7
F1-Score(%)	96.4	77.2	93.3	79.2	90.4	93.3	93.7	93.1	78.7	83.9	76.2	77.5	86.1
**MODEL: DenseNet201**													
Accuracy(%)	99.7	99.4	99.4	100	100	99.1	99.7	99.4	100	99.7	97.6	98.2	99.4
Precision(%)	100	96.6	96.6	100	100	96.3	96.8	100	100	96.8	81.8	92.9	96.5
Recall(%)	96.6	96.6	96.6	100	100	92.9	100	93.3	100	100	93.1	86.7	96.3
F1-Score(%)	98.3	96.6	96.6	100	100	94.6	98.4	96.5	100	98.4	87.1	89.7	96.4
**MODEL: MobileNetv2**													
Accuracy(%)	98.5	97.0	98.8	96.7	99.7	99.7	99.1	99.1	98.2	98.8	96.1	97.9	98.3
Precision(%)	100	88.0	90.3	75.0	93.3	100	96.6	96.6	96.2	96.4	70.0	92.6	91.3
Recall(%)	82.8	75.9	96.6	93.1	100	96.4	93.3	93.3	83.3	90.0	96.6	83.3	90.4
F1-Score(%)	90.6	81.5	93.3	83.1	96.5	98.2	94.9	94.9	89.3	93.1	81.2	87.7	90.4
**MODEL: our model**													
Accuracy(%)	100	99.7	99.1	99.7	100	99.7	99.7	100	100	99.4	98.2	98.5	99.5
Precision(%)	100	100	96.4	96.7	100	100	96.8	100	100	93.8	89.7	93.1	97.2
Recall(%)	100	96.6	93.1	100	100	96.4	100	100	100	100	89.7	90.0	97.2
F1-Score(%)	100	98.3	94.7	98.3	100	98.2	98.4	100	100	96.8	89.7	91.5	97.2

### Feature visualization

**Figure 10:**
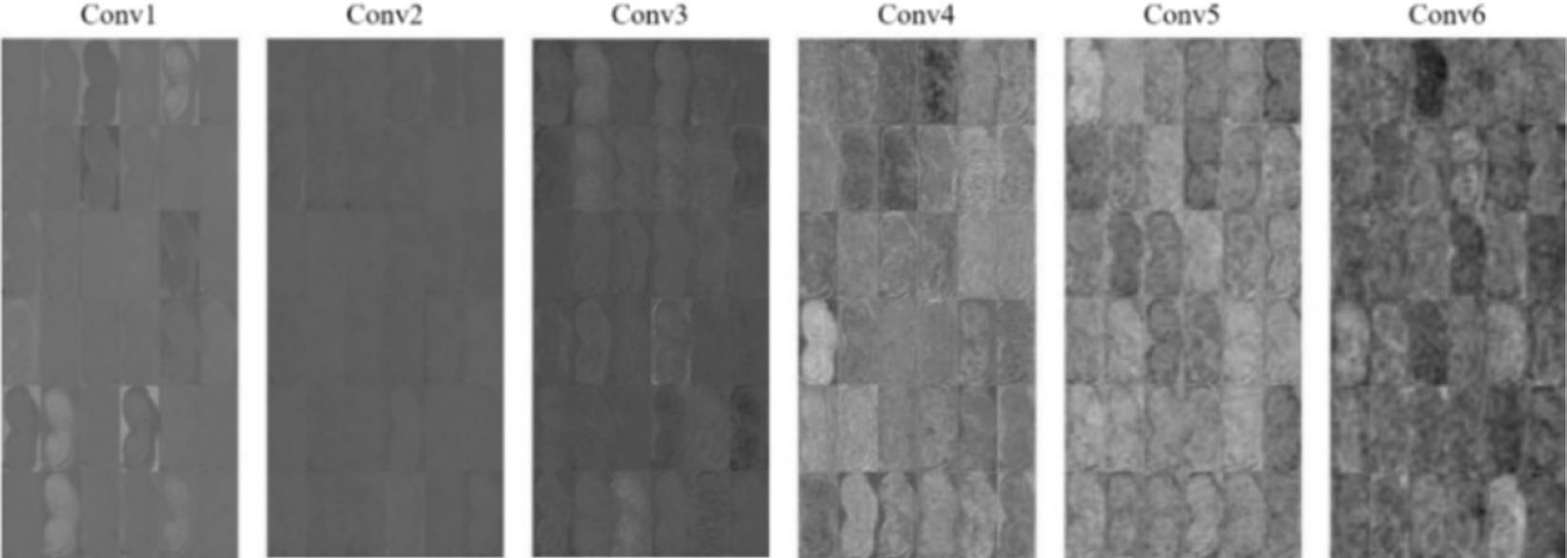
Visualize the image activation region.

This section shows the feature visualization of a convolutional neural network based on Our Model. When the image is input into the convolutional neural network, there will be different activation regions at various network layers. By establishing the comparison between the activation regions at different layers and the original image, network learning characteristics in this layer can be visualized. Figure 10 takes the 101 Huasheng sample as an example to show the activation regions at different layers of Our Model. It can be seen from Fig. 10 that the shallow network will learn simple features such as texture and edge of the image, while the more profound the network is, the more complex and abstract the features it learns.

**Figure 11:**
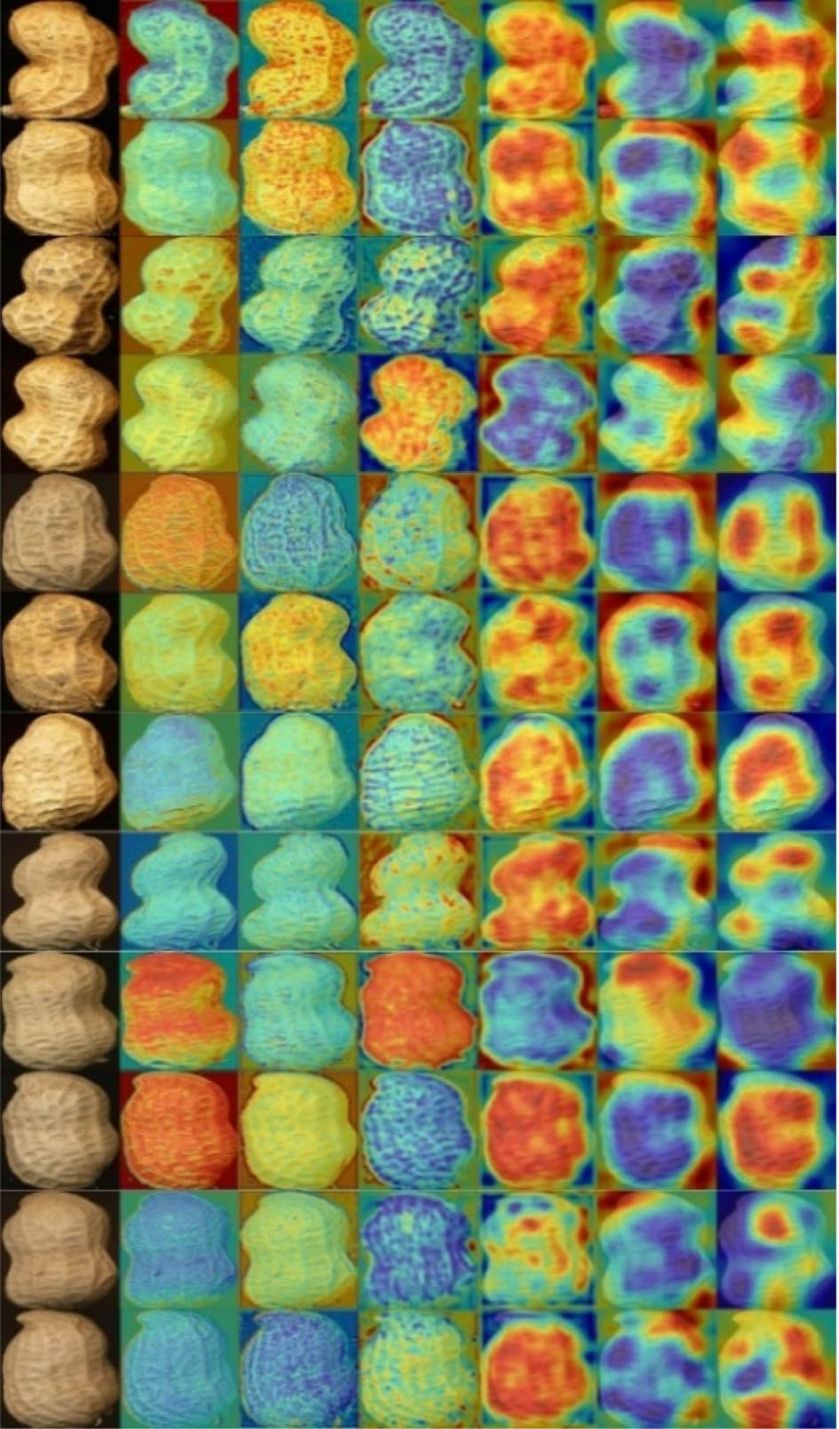
Visualization of Grad-CAM features of our model (From top to bottom are peanut samples of 12 varieties and from left to right are peanut samples and characteristic map of conv1-6).

Gradient-weighted class-activation mapping Grad-CAM^31^ is a method for feature visualization in the class-activation heat map. Grad-CAM can calculate the identification gradient of the final convolution feature map. The larger the gradient is, the more dependent the classification is, and it is represented as the strongly activated region on the feature map. Figure 11 shows the Grad-CAM visualization of all peanuts varieties in the six convolutional layers of Our Model. As shown in Fig. 11, the feature map's red region represents the vital activation region for the network model to identify peanut pod species. In contrast, the blue region represents the weak activation region for the network model to identify peanut pod species. The larger the gradient is, the redder the color of this region will be, and the more potent its influence on the classification result will be. In the identification and classification of peanut pods, the model initially focused on peanut varieties' different textures. With the deepening of network layers, the advanced features of the image were activated, and finally, the vital activation region was located at the mouth and waist of the peanut pod.

**Figure 12:**
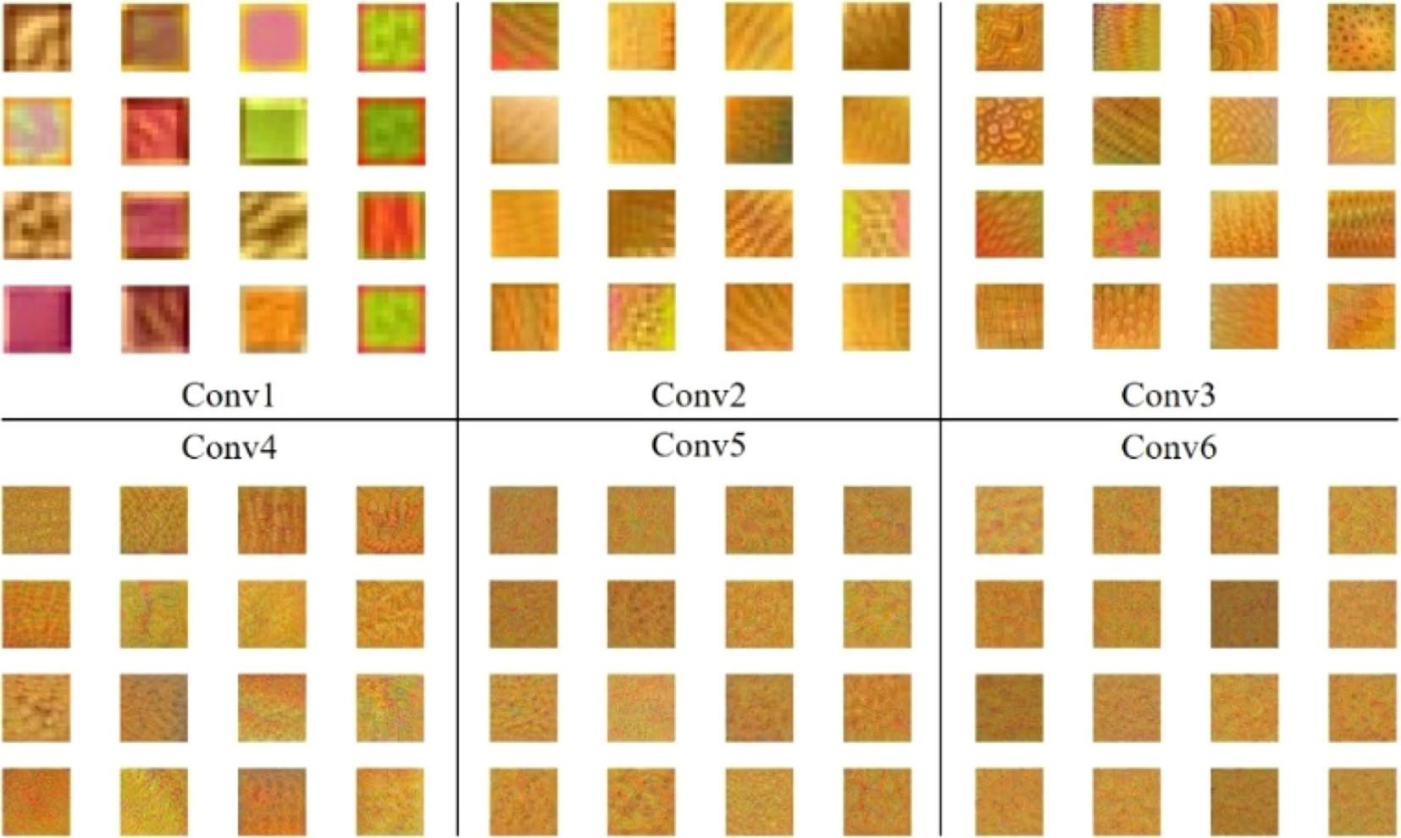
Features captured by our model.

Different layers of the convolutional neural network have different activation regions, so the image features extracted by different layers are also various. As the number of layers deepens, detailed features will decrease and more abstract advanced features will increase^32^. Figure 12 for our six convolutional layers of the model to extract the characteristics of the figure, each layer took 16 characteristics to show, from Fig. 12 intuitive see conv1 learned some image color and contour feature, conv2–conv4 to extract features for image texture, more in conv5 conv6, has the characteristics of a given in the more complex and abstract, difficult to use the human mind to judge, eventually become the advanced features of the model.

### Model applicability testing

**Figure 13:**
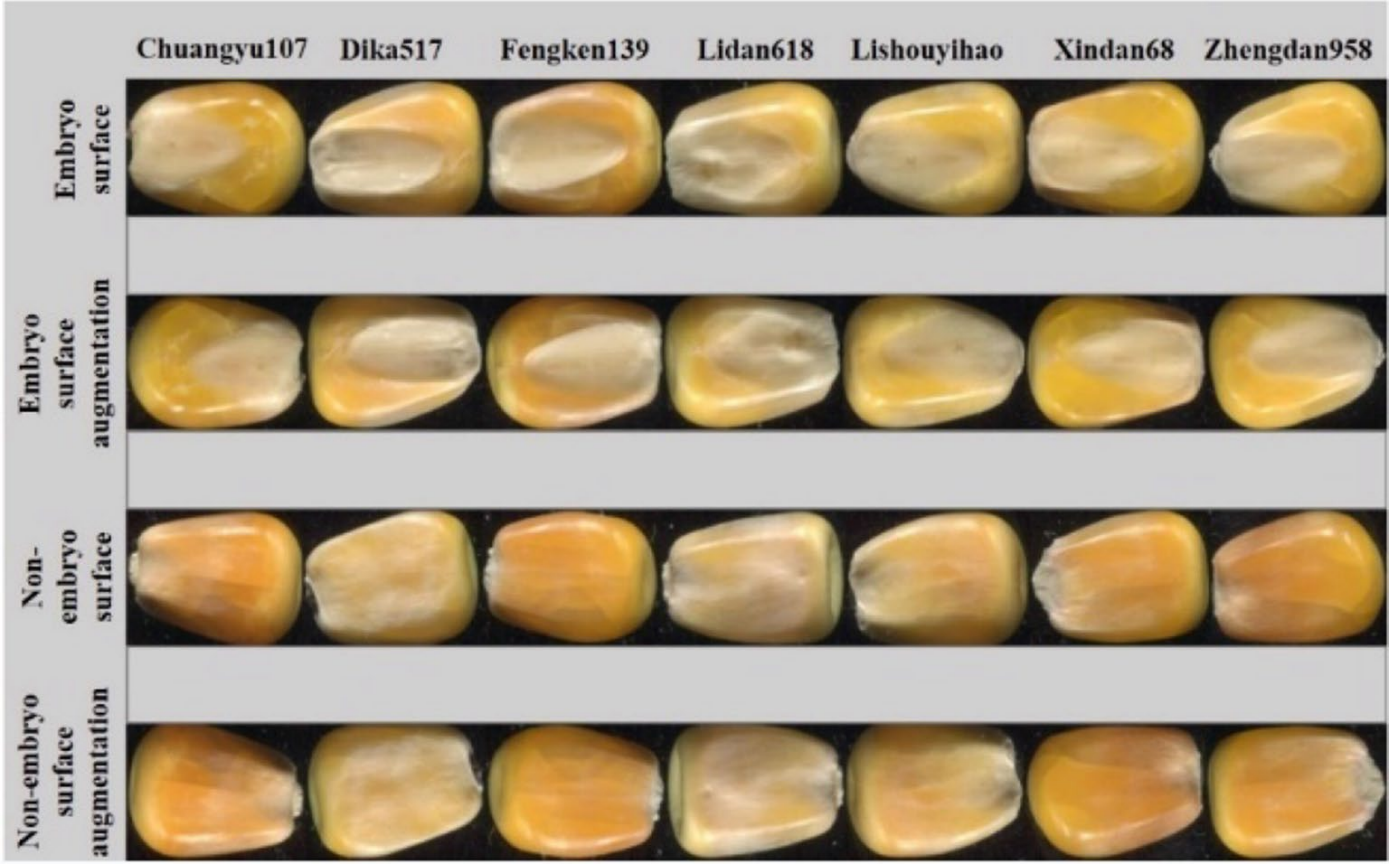
Model applicability test sample.

Our Model was applied to the identification and classification of seven varieties of corn grains to test the model's applicability and prove the robustness and versatility of the improved VGG16 model. The acquisition environment of corn kernel images was the same as that of peanut pods. A total of 1260 corn kernel images of 7 varieties were collected. The data augmentations method was used to expand the image to 2520 images to form the corn kernel dataset. Figure 13 is the sample diagram of the applicability test of this model.

The corn kernel data set was imported into Our Model for the training test and the training process was consistent with the peanut pod identification. The training parameters were set Image Input Size: 224 × 224 × 3;Mini BatchSize: 16; Initial Learn Rate: 1e−4; Validation Frequency: 64. All models finish training after 50 Epochs. The experimental results are shown in Table 8. The model achieved the highest accuracy of 95.63% and the average accuracy of 94.71% on the validation set. The model achieved the highest accuracy of 92.5% and the average accuracy of 90.1% on the test set. The results showed that, although the grain features of corn kernels were more difficult to identify than those of peanut pods, the model still achieved high accuracy, and Our Model was competent for identifying seven types of corn kernels.

**Table 8:** Model applicability test results.

Times	Validation accuracy(%)	Test accuracy(%)
First	93.25	88.5
Second	95.63	92.5
Third	95.24	89.3
Average	94.71	90.1

To further prove Our Model's ability applied to the identification task of seven types of corn kernels, the test model's performance evaluation was added. Table 9 confusion matrix table for identification of maize grain, by the Table 9, the average Accuracy model reached 98.2%, the average precision reached 92.9%, the average Recal reached 92.4%, the comprehensive evaluation index of F1-Score the highest Score of 96%, the average of 92.2%. That result means that the model has a stable and excellent performance, excellent finish seven varieties of corn kernels of work. The results show that Our Model has a good performance in the model applicability test, which proves the robustness and generality of the improved VGG16 model and expands the possibility of the improved VGG16 model applied in the field of Crop variety identification and classification.

**Table 9:**
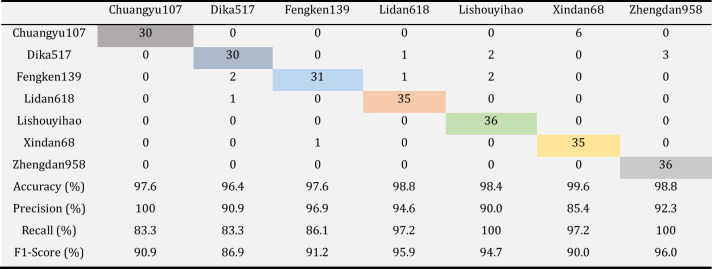
Model applicability test Confusion matrix.

## Discussion

In this paper, the improved VGG16 model was applied to realize the identification and classification of 12 types of peanut pods, consistent with the research direction of literature^3,7^. New technologies are used to solve classical problems, and deep learning technology is introduced into peanut identification and classification to obtain more intelligent and accurate results. Such problem-solving methods are similar to those in literature^20,21^. This paper using the advantages of an advanced convolutional neural network for reference; the improved VGG16 model is improved based on the VGG16 model to achieve a better identification effect. This idea of improving model performance through network improvement is as advanced as literature^33,34^. In this paper, VGG16 was improved by an innovative network improvement method, and the model was introduced into the field of peanut variety identification and classification. The experimental results show that the network improvement method in this paper is effective, and it is feasible to apply the improved network to the field of peanut variety identification.

In the process of network model improvement, the influence of different network improvement methods on the network model's identification effect is compared and discussed. Table 4 shows that Model1 and Model2 have different identification effects due to the BN layer's different placement. The BN layer of Model1 is placed between the convolutional layer and the ReLU layer of the model, while the BN layer of Model2 is placed between the ReLU layer and the Pooling layer of the model. The experimental results show that Model2 achieves an average test accuracy of 96.7%, 2.8% higher than Model1, indicating that Model2 has a better identification effect than Model1. In this experiment, it is better to place the BN layer between the ReLU layer and the Pooling layer, consistent with the reference^28^.

The number of filters in conv6 will affect the performance of the model. The results are shown in Table 5. Model (128) achieved an average accuracy of 95.73% on the validation set and 96% on the test set. Model (256) had an average accuracy of 1.79% higher on the validation set than Model (128). The average accuracy on the test set was 0.2% higher than the model (128). Model (512) had an average accuracy of 0.1% higher on the validation set than Model (256). The average accuracy rate on the test set was 0.5% higher than the model (256). The results show that the model's identification accuracy increases with the orderly increase of the number of filters set in conv6.

The experimental object of peanut variety identification work is the peanut pod. However, the researchers' experimental object in literature^8^ and literature^18^ was peanut seeds. Compared with them, this paper has unique advantages. First of all, in peanut products, peanut pods need to be shelled to obtain peanut seeds. To avoid waste caused by peanut shelled, enterprises will prioritize the sorting of peanut pods. Secondly, compared with the peanut pod, the peanut seeds' sorting process can easily bring pollution and damage to the peanut seeds and reduce the peanut's quality. Besides, the improved VGG16 model in this paper has robustness and applicability for crop identification and classification and it will also achieve good results if applied to peanut seed sorting.

In this paper, the improved VGG16 model was used to identify and classify peanuts, and the algorithm part of the peanut variety identification engineering task was completed. The following research direction should be the practical engineering application of peanut variety identification. For example, the improved model can be configured in a peanut seed sorter^35^. Besides, the model can be deployed on the cloud to realize real-time online identification of peanut varieties on mobile devices^36^. In the peanut phenotype field, the improved model can be applied to high-throughput peanut phenotype detection^37^ to increase peanut phenotype detection accuracy. In terms of breeding, the improved model was applied to the scientific breeding of peanuts^38^ to enhance similar varieties of peanuts' judgment ability. The model is applied to engineering practice to convert the latest technology into consideration economic and social benefits.

## Conclusions

This paper improved the deep convolutional neural network VGG16 and applied the improved VGG16 to the identification and classification task of 12 varieties of peanuts based on deep learning technology. Finally, the average test accuracy was 96.7%, 8.9% higher than that of VGG16. Compared with the classical model, the average test accuracy is 1.6–12.3% higher than other models. In the model applicability test, the average test accuracy is 90.1%. The influence of various model construction methods on the overall performance of the model was compared. It was found that the identification ability of the model would be stronger if the BN layer were placed between the ReLU layer and the Pooling layer. The orderly increase of the number of filters set in the Conv6 layer would improve the model's identification ability. In this paper, deep learning technology was introduced into the field of peanut variety identification. The VGG16 model was successfully improved to obtain the optimal identification effect, which proved the feasibility of a convolutional neural network in the field of crop variety identification and classification. The model improved in this paper has positive significance for exploring other Crop variety identification and classification.
